# Could a phase model help to improve translational animal research?

**DOI:** 10.1002/ame2.12284

**Published:** 2022-10-20

**Authors:** Benjamin Mayer, Jan Tuckermann, Rainer Muche

**Affiliations:** ^1^ Institute of Epidemiology and Medical Biometry Ulm University Ulm Germany; ^2^ Institute of Comparative Molecular Endocrinology Ulm University Ulm Germany

**Keywords:** confirmatory trials, hypothesis testing, results interpretation, transferability

## Abstract

**Background:**

Animal models are widely applied in medical research for different purposes. In particular, results from translational experiments may be used for subsequent clinical development. However, transferability of these findings to the human organism is controversial. Among other factors, this may be traced back to a lack of clear differentiation of the evidence (explorative vs. confirmatory) provided by such experimental results. In general, inferential statistics (i.e. *p* values) should not be interpreted in as confirmatory unless crucial methodological requirements are met.

**Methods:**

Therefore, we propose a phase model which reflects the well‐established process of clinical research, and we discuss its potential to improve decision making in translational research. The model aims to clarify the reliability of results derived from animal models.

**Results:**

The phase model proposes subdividing translational, pre‐clinical research into pilot, exploration, and confirmation phases. Experiments for which there is no valid estimation of the expected effect size are designated as pilot studies. Based on these data, experiments in subsequent phases may be planned using both appropriate design and statistical methods.

**Conclusion:**

Separating the entire process of translational animal research into three phases could contribute to improved transparency of the evidence derived from such experiments.

## Graphical abstract

Transferability of findings from animal research to the human organism is controversially discussed. We propose a phase model which refers to the well‐known process of clinical research in order to improve decision‐making regarding the evidence which is gained in translational animal research. The model suggests subdividing translational, pre‐clinical research into a pilot, exploration, and confirmation phase.

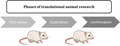

## 1 INTRODUCTION

Despite progress in technical developments of in vitro and in silico experimental approaches, animals are still commonly used in medical research, e.g. in basic research experiments, for toxicological screenings, for mechanistic studies using genetically modified organisms with gain/loss of functions or functional knock‐ins, or for translational animal studies. The latter focus aims at exploring novel approaches (derived from basic science) for either treating or preventing diseases. It is commonly acknowledged that this will remain unchanged anytime soon,^1^, ^2^ although transferability of results is often called into question.^3^ Debate regarding this problem of transferability is associated with insufficient information provided by published authors, poorly planned experiments, and inconsistent implementation of available guidelines for reporting on in vivo experiments.^4^, ^5^, ^6^, ^7^, ^8^

Utilization of translational animal models is often indispensable due to the complexity of living organisms and the resulting need for in vivo evaluation. All studies, including those with a different research focus as outlined above, follow the 3R principle (*reduction*, *replacement*, and *refinement* of animal studies) of Russell and Burch.^9^ Further, it is standard practice (e.g. due to the EU directive 2010/63/EU)^10^ to justify the number of experimental animals during the application process. At the same time, however, there is no consensus yet on the general validity of statistical results in animal studies,^11^ which leads to the following dilemma.

In order to maximize gain of knowledge, a large number of endpoints are usually assessed in animal trials, but, in contrast to clinical studies, no primary endpoint leading to a confirmatory interpretation needs to be defined. Thus, from the strict perspective of inferential statistics one could argue that the results of animal studies should be interpreted in an explorative manner only. Otherwise, the overall type 1 error (‘α‐level’) is likely to be inflated by multiple hypothesis testing if no adjustment is applied. However, this could contradict the *reduction* aspect of the 3Rs if standard methods of α‐adjustment are used (e.g. Bonferroni) during sample size estimation. On the other hand, translational animal trials should meet the requirements for interpretation as confirmatory results because of their role as a ‘bottleneck’ in translational research, i.e. selection of the most promising hypotheses for clinical development. Regarding pharmaceutical compounds, only those with the best risk–benefit profile (safety and efficacy) should be applied to the human organism. Hence, translational animal studies should go beyond the aspiration of just generating new hypotheses.

It is not appropriate to determine the validity of statistical results principally in animal experiments.^4^ If it is not possible to guess the expected effect in advance, e.g. due to a lack of preliminary data,^12^ no formal calculation of the required animal numbers can be done, so all findings of such ‘pilot studies’ are fully explorative. Confirmatory hypothesis testing is only possible in prospective studies that include a valid sample size estimation, as well as an appropriate approach to multiple hypothesis testing, if necessary. This is analogous to the situation in clinical research, which also involves studies with diverse results in terms of validity of statistical analysis (e.g. single arm phase I trials vs. randomized controlled phase III trials [RCTs]). A comparable approach seeking a transparent structure of interpretation of results has also been implemented for diagnostic studies, defining and explaining the different phases of development of diagnostic measures.^13^ However, the current lack of clear guidance on when animal trial results may be interpreted in a confirmatory manner, and which key requirements with regard to experimental design need to be fulfilled, poses a frustrating problem that gets in the way of the translational process.^14^ A phase model for translational animal research based on the corresponding model from clinical research could help researchers to better evaluate and delimitate the findings of animal studies. Thus, the aim of this article is to propose such a model that takes into consideration the demands of practical translational animal research.

## 2 METHODS

### 2.1 Persisting challenges of translational animal research

The proposed phase model for translational animal research provides a theoretical framework to handle two major challenges in today's practice, and so is able to help improve the quality of results.

First, there is no consensus yet which methodological presumptions must be met in order to reach confirmatory significance in animal trials. Current German, and also European, legislation differentiates between experiments to ‘generate new hypotheses’ and those to ‘prove (established) hypotheses’,^10^ though adequate consideration of a possibly inflated type 1 error due to multiple hypothesis testing is actually not implemented, as it is for instance in clinical studies.^15^ However, this multiplicity issue is common in animal trials because of the large number of endpoints that are usually assessed. Standard approaches to multiplicity adjustment, like Bonferroni,^16^ conflict with the 3R *reduction* aspect of the trials since smaller type 1 error rates per hypothesis would raise the required sample size.^15^

Further, availability of preliminary data varies a lot. For some translational studies there are data with high transferability that can be used for sample size estimation. In contrast, other experiments are considered to be fully exploratory pilot studies since no data are available to calculate the required sample size. The possibility and application of sample size estimation are key presumptions of confirmatory results interpretation, which is why it seems unreasonable to interpret results from translational projects uniformly.

Finally, translational animal studies may launch subsequent research in humans. The most promising findings from such trials in terms of both efficacy and harmlessness may lead to a first‐time use of the tested substances in humans. In contrast, the U.S. Food and Drug Administration (FDA) primarily relies on toxicity studies.^17^ Thus, translational animal studies should go beyond the aspiration of simply generating novel hypotheses. However, this presumes that a primary endpoint (or a set of primary endpoints) is defined, for which the experiment is specifically planned. Compared to the current situation, this corresponds to a paradigm shift to some extent.

### 2.2 Benefit of a phase model

In summary, studies in the field of translational animal research rely on different conditions (data availability, research hypothesis, overall goal of the trial) to those in clinical research. This is why it is difficult generally to interpret the results of these studies in either a confirmatory or explorative manner, and this points to the need for a phase model for preclinical research. An outstanding characteristic of clinical research is the separation of the whole development process. The single phases put emphasis on different aspects of both efficacy and safety of the treatment of interest (phase 1: efficacy and safety in healthy volunteers; phase 2: efficacy and safety in well‐selected patients; phase 3: efficacy and safety in large patient populations using RCTs for approval; phase 4: long‐term safety after approval).^18^ Most important, the application of this phase model enables direct assessment of the value of the findings, especially with respect to the question of whether the results will either generate or prove the underlying hypotheses.

**FIGURE 1:**
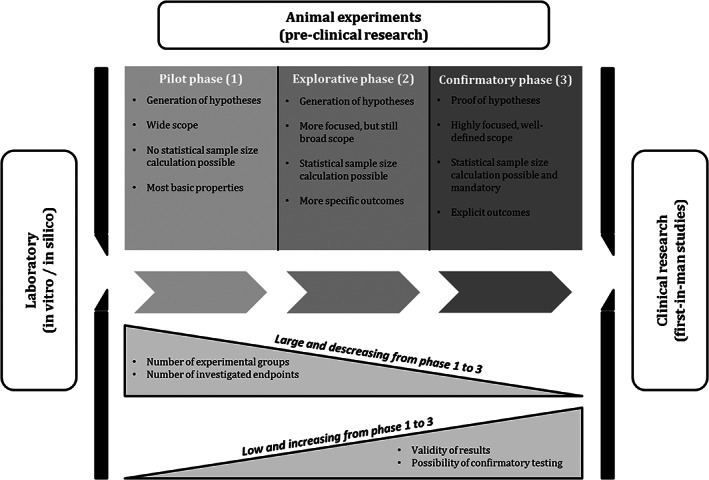
Phases of translational animal research

The major benefit of implementing such a theoretical phase model in animal research will be an increase in transparency, involving interpretation of experimental results as well as requirements to be met for study planning. The particular characteristics of each phase in the proposed model (Figure 1) and the dissociation of the phases from each other enable the principal investigator to clearly define the responsibilities that must be complied with. The characteristics of each phase will also imply the most appropriate way to interpret results. With regard to stronger adherence to the ARRIVE guidelines,^7^ it would be worthwhile establishing the popularly accepted study design features that need to be described when results are published. Well‐designed experiments are also indispensable for the development of appropriate replacement methods.

## 3 RESULTS

The proposed phase model for translational animal research needs, of course, to adequately describe all possible scenarios that may occur when planning such trials. The single phases have to be clearly dissociated, leaving no scope for discussion about which phase a particular study shall be assigned to. To our knowledge, there has as yet been no attempt to separate the different objectives of translational animal studies systematically, although the statistical results validity from pilot studies and experiments that have been planned properly using statistical methods obviously differs. A more rigorous differentiation between explorative and confirmatory experiments has already been proposed in the past.^14^, ^19^, ^20^ Essentially, the proposed phase model suggests distinguishing between pilot studies, explorative studies, and confirmatory trials (Figure 1).

### 3.1 Pilot phase 1

The effect size is a key parameter for statistical sample size calculation. It may be estimated based on preliminary data or, if not available, medical as well as biological considerations can be used to calculate a minimally relevant effect size. If this is information is not available, a statistical sample size calculation cannot be performed. Thus, experiments in this pilot phase 1 are not able to generate confirmatory results, i.e. they need to be interpreted as fully explorative. The pilot phase 1 is however able to reveal new research hypotheses to be addressed in subsequent phases. Usually, these experiments include both a large number of experimental groups and endpoints, but the sample size per group is typically very small. Since standard calculations are not possible, the sample size is commonly chosen pragmatically. Alternatively, simulation approaches can be used. In practice the group size chosen depends on the scale level of the endpoint(s) of interest: commonly used groups sizes range from 5 to 6 animals in the case of continuous endpoints, 7 to 8 animals in case of categorical endpoints, and 10 to 12 animals for time‐to‐event analyses. These numbers turned out to be reasonable choices when using a Markov Chain Monte Carlo (MCMC) simulation approach for verification.^12^ However, in practice group numbers of as little as 3 animals are often reported.

### 3.2 Explorative phase 2

If an effect size of interest can be defined, i.e. statistical sample size estimation is possible, but the number of endpoints is still large and the primary focus is on hypothesis generation, studies should be viewed as part of the exploration phase. Findings from pilot phase 1 studies, among others, may be used to statistically plan such experiments. Usually research questions are more focused compared to the pilot phase 1, but are still broad in scope, e.g. due to a large number of experimental groups or endpoints of primary interest. With regard to statistical hypothesis testing, studies in the explorative phase 2 do not involve the need to adjust the type 1 error level addressing the multiplicity issue. As a consequence, the regular two‐sided α‐level may be applied to all hypotheses, which leads to comparatively small group sizes. If appropriate, further methodological approaches may be implemented in the process of sample size calculation, e.g. resampling methods,^21^ to estimate the group size correctly and in accordance with the 3Rs.

### 3.3 Confirmatory phase 3

The final phase 3 focuses on a confirmatory proof of pre‐specified research hypotheses and a well‐defined scope of the experiment. This phase can fall back on extensive findings from both preceding phases, so a prioritization of the most relevant endpoints is feasible. Statistical estimation of the required sample size is possible, as well as mandatory, in order to ensure confirmatory results interpretation. Moreover, in the case of multiple research hypotheses the type 1 error level requires adjustment in order to meet confirmatory demands. At this stage, the application of the most commonly used α‐error adjustment methods, like Bonferroni,^16^ should be avoided if possible because of their tendency to decrease the α‐error applied to statistical sample size estimation. In order to adhere to the 3R principle aiming at small group sizes, alternative measures that deal with the multiple comparisons problem without explicitly adjusting the α‐level (e.g. hierarchical testing, gatekeeping^22^) may be applied. Moreover, further standard elements of clinical studies, e.g. randomization and blinding, should be implemented as far as possible in final phase 3 animal research studies to obtain the highest possible level of statistical results validity.^23^, ^24^

### 3.4 Application example

In order to demonstrate the consequences of the proposed phase model, one may consider an example animal trial following a 3 × 2 design. It is assumed that there are three interventional groups treated with different substances (experimental substance 1 (ES1), experimental substance 2 (ES2), and control substance (CS)) in mice of two different genotypes (mutant and wild‐type) with the aim of assessing their impact on a continuous outcome variable (cytokine concentration [CC]).

In the case when there are no preliminary data on CC (and also no relevant effect size can be defined from a medical perspective), a statistical calculation of the required sample size is not possible. Therefore, in accordance with the proposed model in Figure 1, this study needs to be treated as a pilot phase 1 experiment. Since statistical calculation of the required sample size is impossible in this situation, group sizes must be determined in accordance with the suggestions for continuous endpoints from MCMC simulation, i.e. a group size of *n* = 5 animals could be an initial guess.^12^

If a CC‐related effect size can be reasonably defined, statistical sample size estimation is possible. According to the phase model it is crucial to decide whether the focus is still on hypotheses generation (phase 2) or confirmation (phase 3). The latter would require (i) defining which hypotheses require a confirmatory result, and thus (ii) the measures to be taken to address the issue of multiple testing, if necessary. To demonstrate the importance of distinguishing between the explorative and the confirmatory phase, it should be assumed that there is some information on CC data for the underlying 3 × 2 design (Table 1). Assuming normally distributed data, either an ANOVA‐based plan or a series of *t* tests covering all relevant pairwise comparisons may be used. In the case of the ANOVA approach either a one‐ or two‐factorial setting is possible. This depends on the primary objective of the study, i.e. showing differences in treatment groups, genotypes, or a combination of both.

**TABLE 1:** Group means (standard deviations) of cytokine data in different treatment groups and genotypes

Treatment group	Genotype		Σ
	Wildtype	Mutant	
ES1	6.1 (1.7)	7.5 (2.4)	6.8 (2.1)
ES2	5.3 (1.5)	5.9 (1.5)	5.6 (1.5)
CS	9.1 (0.9)	13.4 (2.9)	11.3 (3.1)
Σ	6.8 (2.1)	8.9 (4.0)	7.9 (3.3)

Abbreviations: CS, control substance; ES1, experimental substance 1; ES2, experimental substance 2.

In any case it is mandatory to allocate the trial either to the explorative phase 2 or the confirmatory phase 3, since this has a major impact on the assumed type 1 error during the sample size estimation process. In Table 2 the resulting sample sizes are summarized. All calculations relied on common assumptions of a two‐sided type 1 error of 5% and a power of 80%, whereas a possible required adjustment of the overall 5% α‐level would follow a Bonferroni approach. If the underlying 3 × 2 study design had been planned as a pilot experiment, group sizes probably would not have exceeded *n* = 5 animals resulting in 30 animals in total. Assuming this study belongs to the explorative phase, group sizes would range from *n* = 4 to *n* = 13 animals using a two‐way ANOVA approach, with an even higher range of sample sizes required in the case of a one‐way ANOVA and *t* test‐based planning. If the aim is to demonstrate a treatment effect it seems reasonable to apply for *n* = 10 animals per group in order to get a significant result for both pairwise comparisons ES1 vs. CS and ES2 vs. CS, respectively. Using the one‐way ANOVA approach, i.e. *n* = 4 animals per group, would very likely lead to a power problem for the pairwise comparisons. Therefore, the total sample size for a phase 2 animal trial would be around 60 animals. Finally, the group size would be slightly increased if this sample study aimed to achieve confirmatory proof of both treatment‐related pairwise comparisons. Using a Bonferroni adjustment of *α* = 0.025 would lead to a reasonable group size of *n* = 12 animals. Thus, the overall sample size would increase to 72 animals.

**TABLE 2:** Estimated group sizes for cytokine data with respect to the independent variables “treatment group” and “genotype”

Calculation approach		Treatment group		Genotype
ANOVA	Two‐way^a^	4		13
	One‐way	4		40
*t* test	No *α*‐adjustment^b^	ES1 vs. CS	10	
		ES2 vs. CS	7	
		ES1 vs. ES2	120	
	Including *α*‐adjustment^b^	ES1 vs. CS	12	–
		ES2 vs. CS	8	
		ES1 vs. ES2	120	

Abbreviations: CS, control substance; ES1, experimental substance 1; ES2, experimental substance 2.

^a^ *α*‐adjustment following the Bonferroni method, whereas the confirmatory focus is on superiority of any experimental substance against control substance (two pairwise comparisons), so *α* = 0.025 for ES1 vs. CS and ES2 vs. CS; *α* = 0.05 for ES1 vs. ES2 in an explorative setting;

^b^ At least 80% power for both treatment group as well as genotype.

## 4 DISCUSSION

Animal experiments are still an indispensable component of basic medical research, although on the one hand transferability of results from animal models to research in humans has been questioned and on the other the need for more stringent criteria of statistical results validity has been emphasized.^25^, ^26^, ^27^ Thus, improvements in the methodological strategy of planning translational animal trials are urgently required. These concerns both general study design aspects as well as appropriate statistical methods for data analysis.^24^ Despite the fact that according to the ARRIVE guideline it is actually not essential to focus on reporting *p* values when interpreting the results from animal studies, this is still the most common way to present results. Although the usage of *p* values is controversial, they are a well‐accepted and established measure of statistical analysis.^28^ However, with the aim of more comprehensive planning and analysis of animal studies there has to be even more emphasis on differentiating between explorative and confirmatory data analysis. Only the latter provides a statistical proof of predefined research hypotheses, and requires the use of statistical sample size estimation techniques beforehand. In particular, appropriate adjustment methods to address the multiple testing problem have to be implemented in the case of multiple endpoints of primary interest.

The proposed phase model for translational animal research is meant to provide a conceptual framework which may help to increase clarity when interpreting results. It must not be understood as an additional regulatory obstacle in practice. Applying the proposed phase model could lead to a more transparent dissociation of findings, involving different levels of statistical results validity. This might help mediate the existing disagreements about the scientific value of animal studies. The phase model could also help to define more precisely the unique standards and requirements of the experiments involved – as well as their implementation in daily routine. Researchers would be able directly assess the most important requirements of each singe phase using the scheme presented in Figure 1. Referencing reported results of an animal trial to the phases presented here would help to clearly appraise their value. With respect to the aim of reducing the number of animals used in medical research, the limitation of smaller sample sizes in the first two phases might have an indirect effect on the whole research process. As in the case of early phase 1 trials in humans focusing on pharmacokinetics, one might consider dispensing with adherence to common criteria of clinical studies, e.g. a strict type 1 error level of 5%, in order to save additional animals.^24^, ^29^, ^30^

With respect to adhering to the proposed phase model in practice, one might call into question whether it is mandatory to always run through all 3 phases of the framework, or whether e.g. the explorative phase 2 might be skipped. This would, of course, decrease the total sum of experimental animals and consequently contribute to the *reduction* aspect of the 3R principle as well as accelerating the development process. Although this seems worthwhile in some situations with a precisely defined research goal (e.g. identification of an effective vaccination approach for Sars‐CoV‐2), it would be contrary to the aim of increasing reproducibility in animal research as requested by, among others, the ARRIVE 2.0 update.^31^ The reproducibility crisis is, of course, multifaceted and also concerns other aspects of transparency (e.g. omitting data) and trial design (e.g. double‐blinding, randomization). On the other hand, the proposed phase model is intended to assist researchers in sharpening their project aims with the overall aim of increasing transparency. Thus, the phase model must not be misinterpreted as an additional obstacle in the process of animal experiments, and for particular projects it could be sufficient to run only selected phases of the proposed model.

Based on the example of the ARRIVE initiative, it is obvious that implementation of proposed guidelines can hardly be realized if not challenged consequently in practice.^5^ The actual implementation of the proposed phase model in daily practice can only be enforced and promoted with the help of approving authorities. Thus, should the presented phase model be accepted, as a first and most important consequence, the model needs to be considered in the official application forms and regulation processes. Researchers will need to be encouraged to assign their projects to a particular phase. At the same time, the benefits of the proposed model have to be explained to researchers and relevant journals in the field, as has been successfully done in promoting the ARRIVE guidance^7^ for animal research or the CONSORT statement^32^ for clinical studies. The implementation of the proposed concept also needs to be accompanied by an evaluation of the strengths and limitations of its current version. Given this outcome, the proposed phase model could help to increase transparency in animal trials, and to better structure the whole process of pre‐clinical research.

## AUTHOR CONTRIBUTIONS

Benjamin Mayer and Rainer Muche developed the idea for the proposed phase model; Benjamin Mayer drafted the manuscript, Jan Tuckermann and Rainer Muche critically reviewed the initial draft and suggested revisions and extensions. All authors agreed with the submitted, final form.

## CONFLICT OF INTEREST

All authors declare that they have no potential conflicts of interest with respect to this submission.

## References

[1] Bresson D , von Herrath M . Humanizing animal models: a key to autoimmune diabetes treatment. Sci Transl Med. 2011;3:68ps4. 10.1126/scitranslmed.3002102 PMC3137650 21289271

[2] Lloyd KCK , Robinson RN , MacRae CA . Animal‐based studies will be essential for precision medicine. Sci Transl Med. 2016;8:352ed12. 10.1126/scitranslmed.aaf5474 27535618

[3] Rosenblatt M . The large pharmaceutical company perspective. N Engl J Med. 2017;376:52‐60. 28052221 10.1056/NEJMra1510069

[4] Baker M . Missing mice: gaps in data plague animal research. Nature. 2016. doi:10.1038/nature.2015.19101

[5] Baker D , Lidster K , Sottomayor A , Amor S . Two years later: journals are not yet enforcing the ARRIVE guidelines on reporting standards for pre‐clinical animal studies. PLoS Biol. 2014;12:e1001756. 24409096 10.1371/journal.pbio.1001756 PMC3883646

[6] Cressey D . Poorly designed animal experiments in the spotlight. Nature. 2015. doi:10.1038/nature.2015.18559

[7] Percie du Sert N , Ahluwalia A , Alam S , et al. Reporting animal research: explanation and elaboration for the ARRIVE guidelines 2.0. PLoS Biol. 2020;18(7):e3000411. 32663221 10.1371/journal.pbio.3000411 PMC7360025

[8] Macleod MR . Preclinical research: design animal studies better. Nature. 2014;510:35. doi:10.1038/510035a 24899295

[9] Russell WMS , Burch RL . The Principles of Humane Experimental Technique. Methuen; 1959.

[10] European Union . Directive 2010/63/EU of the European Parliament and of the council of 22 September 2010 on the protection of animals used for scientific purposes. Off J Eur Union. 2010;L276/33‐L276/79.

[11] Gaus W , Mayer B , Muche R . Interpretation of statistical significance—a discussion on exploratory versus confirmative testing in clinical trials, epidemiological studies, meta‐analyses, and toxicological screening. J Clin Exp Pharmacol. 2017;5(4):1000182.

[12] Allgoewer A , Mayer B . Sample size estimation for pilot animal experiments by using a Markov chain Monte Carlo approach. ATLA. 2017;54:83‐90. 10.1177/026119291704500201 28598193

[13] Koebberling J , Trampisch HJ , Windeler J . Memorandum for the Evaluation of Diagnostic Measures (in German). Schattauer; 1989.

[14] Kimmelman J , Mogil JS , Dirnagl U . Distinguishing between exploratory and confirmatory preclinical research will improve translation. PLoS Biol. 2014;12(5):e1001863. 24844265 10.1371/journal.pbio.1001863 PMC4028181

[15] European Medicines Agency . Guideline on Multiplicity Issues in Clinical Trials. European Medicines Agency; 2017.

[16] Dunn OJ . Multiple comparisons among means. JASA. 1961;56(293):52‐64. doi:10.1080/01621459.1961.10482090

[17] U.S. Department of Health and Human Service, Food and Drug Administration (FDA) . Guidance for Industry: M3(R2) Nonclinical Safety Studies for the Conduct of Human Clinical Trials and Marketing Authorization for Pharmaceuticals. Food and Drug Administration (FDA); 2010.

[18] DeMets D , Friedman L , Furberg C . Fundamentals of Clinical Trials. 4th ed. Springer; 2010.

[19] Howells DW , Sena ES , Macleod MR . Bringing rigour to translational medicine. Nat Rev Neurol. 2014;10:37‐43. 24247324 10.1038/nrneurol.2013.232

[20] U.S. Department of Health and Human Service, Food and Drug Administration (FDA) . Guidance for Industry: Drug Interaction Studies—Study Design, Data Analysis, Implications for Dosing, and Labelling Recommendations (Draft Guidance). FDA; 2012.

[21] Gietz L , Mayer B . A resampling approach for sample size estimation in animal experiments. J Int Transl Med. 2017;5(2):53‐62.

[22] Mayer B , Stahl V , Kron M . Statistical planning of animal experiments using gatekeeping procedures. ATLA. 2017;45:317‐328. 29313703 10.1177/026119291704500608

[23] Hirst JA , Howick J , Aronson JK , et al. The need for randomization in animal trials: an overview of systematic reviews. PLoS ONE. 2014;9(6):e98856. 24906117 10.1371/journal.pone.0098856 PMC4048216

[24] Strech D , Dirnagl U . 3Rs missing: animal research without scientific value is unethical. BMJ Open. 2019;3:e000035. doi:10.1136/bmjos-2018-000048 PMC8647585 35047678

[25] Anders HJ , Vielhauer V . Identifying and validating novel targets with in vivo disease models: guidelines for study design. Drug Discov Today. 2007;12:446‐451. 17532528 10.1016/j.drudis.2007.04.001

[26] Bhogal N , Balls M . Translation of new technologies: from basic research to drug discovery and development. Curr Drug Discov Technol. 2008;5:250‐262. 18690893 10.2174/157016308785739839

[27] Shanks N , Greek R , Greek J . Are animal models predictive for humans? Philos Ethics Humanit Med. 2009;4:2. 19146696 10.1186/1747-5341-4-2 PMC2642860

[28] Wellek S . A critical evaluation of the current “*p*‐value controversy”. Biom J. 2017;59(5):854‐872. 28504870 10.1002/bimj.201700001

[29] Richter V , Muche R , Mayer B . How much confidence do we need in animal experiments? Statistical assumptions in sample size estimation. J Appl Anim Welf Sci. 2018;21:325‐333. doi:10.1080/10888705.2018.1423972 29370714

[30] Schuirmann DJ . A comparison of the two one‐sided tests procedure and the power approach for assessing the equivalence of average bioavailability. J Pharmacokinet Biopharm. 1987;15:657‐680. 3450848 10.1007/BF01068419

[31] du Sert NP , Hurst V , Ahluwalia A , et al. The ARRIVE guidelines 2.0: updated guidelines for reporting animal research. PLoS Biol. 2020;18(7):e3000410. doi:10.1371/journal.pbio.3000410 32663219 PMC7360023

[32] Schulz KF , Altman DG , Moher D , for the CONSORT Group . CONSORT 2010 statement: updated guidelines for reporting parallel group randomised trials. BMJ. 2010;340:c332. 20332509 10.1136/bmj.c332 PMC2844940

